# Transforming growth factor-β enables NFATc1 expression during osteoclastogenesis

**DOI:** 10.1016/j.bbrc.2007.11.120

**Published:** 2008-02-01

**Authors:** S.W. Fox, K.E. Evans, A.C. Lovibond

**Affiliations:** a School of Biological Science, Ecotoxicology and Stress Biology Group, Room 404 Davy, University of Plymouth, PL4 8AA, UK; b Department of Cellular Pathology, St. George’s Hospital, SW17 0RE, UK

**Keywords:** Osteoclast, TGF-β, Priming, NFATc1, Monocyte differentiation

## Abstract

Osteoclastogenesis is dependent on distinct stimuli that prime and activate osteoclast differentiation. One cytokine needed to prime monocytes for osteoclastogenesis is TGF-β, which enables and augments RANKL and TNF-α-induced osteoclast differentiation. However, the precise time-period during which this occurs and the molecular mechanism mediating this action are unknown. We report here TGF-β prime monocytes for osteoclast formation within 24 h by regulating expression of NFATc1, a key osteoclastic transcription factor. TGF-β directly induces cytoplasmic NFATc1 expression within 24 h, but is unable to stimulate NFATc1 nuclear translocation. Furthermore, RANKL-induced NFATc1 expression is dependent on the presence of TGF-β during the early stages of osteoclastogenesis. Similarly, TNF-α activates osteoclastogenesis by stimulating translocation of TGF-β-induced NFATc1. In light of these findings, it is apparent that osteoclast formation is dependent on coordinated interactions between TGF-β and RANKL/TNF-α that regulate the expression and intracellular distribution of NFATc1 during early stages of osteoclast differentiation.

Bone is optimized for the skeleton’s mechanical and mineral storage roles. Changes in physical activity or circulating calcium elicit a remodeling response that maintains structural competency or restores serum calcium. This is mediated by osteoblasts, which synthesize new bone, and osteoclasts, which resorb bone. It is critical that osteoclast formation is tightly regulated as excessive resorption can cause disorders such as osteoporosis.

Activation of osteoclast differentiation from monocytic precursors is dependent on a balance between levels of the osteoblast-derived cytokine, receptor activator of nuclear factor-κB ligand (RANKL) and its soluble decoy receptor, osteoprotegerin (OPG). Resorptive stimuli increase RANKL and suppress OPG expression to activate osteoclast formation [1,2]. Subsequent binding of RANKL to its receptor RANK induces osteoclastic gene transcription via signaling intermediates including nuclear factor-κB (NFκB), nuclear factor of activated T cells c1 (NFATc1), and AP-1 (see [3]).

Alongside RANKL, monocytes require further bone-derived inputs to prime and enhance their response to RANKL. Without this enabling input osteoclastogenesis will not occur. This is supported by the observation that RANK expressing monocytes do not form osteoclasts outside of bone even in the presence of RANKL at doses far greater than needed to induce differentiation *in vitro* [4]. One key enabling factor is transforming growth factor-β (TGF-β), which primes and enhances the response of monocytes to RANKL or TNF-α [4–9]. Precursors failing to receive this priming stimulus will not develop into osteoclasts [4–6]. This facilitative action is in part achieved by antagonizing the anti-osteoclastic action of inflammatory cytokines, which would otherwise prime precursors to macrophage lineages [10,11]. However, this does not represent the sole mechanism by which TGF-β facilitates and enhances osteoclastogenesis, as TGF-β also augments osteoclast formation in the absence of inflammatory cytokines [5,9].

TGF-β and RANKL utilize overlapping intracellular signaling cascades [3]. These regulate osteoclastic gene expression via key transcription factors such as NFATc1 and *c-fos*. Like TGF-β, these transcription factors are essential for osteoclast formation [12,13]. It is possible, therefore, that TGF-β’s enabling and augmentative effect could be mediated via an action on these transcription factors. To investigate this, it was first necessary to determine the time-period during which TGF-β enables osteoclastogenesis, and establish whether TGF-β-induced signals are essential at the inception of osteoclastogenesis or necessary throughout the differentiation process. Once this had been established, the ability of TGF-β to modify NFATc1 and *c-fos* expression during this period was examined.

## Materials and methods

*Media and reagents.* Stroma depleted nonadherent, M-CSF dependent bone marrow monocytes (BMM) and RAW264.7 murine monocytes (ATCC, UK) were incubated in MEM and Earle’s salts (EMEM) supplemented with 10% FCS (Autogen Bioclear, UK), 2 mmol/l glutamine, 100 IU/ml benzylpenicillin, and 100 mg/ml streptomycin (all from Sigma, UK). Incubations were performed at 37 °C in 5% CO_2_, and cultures fed every 2–3 days. Recombinant human M-CSF, soluble human recombinant RANKL, recombinant human OPG, and murine recombinant TGF-β1 were obtained from Insight Biotechnology. Anti-TNF-α antibody and pan-specific TGF-β antibody were purchased from R&D systems. Anti-NFATc1 antibody was obtained from Santa Cruz Biotechnology. All other reagents were obtained from Sigma unless stated.

*Isolation of BMM.* Female MF-1 mice (4–6 weeks old) were killed by cervical dislocation. Femur and tibia were removed and dissected free of soft tissue. The bone ends were cut and marrow flushed out with medium 199. Cells were washed, resuspended in EMEM, and incubated for 24 h in M-CSF (5 ng/ml) at a density of 3 × 10^5^/ml. After 24 h, BMM were harvested, washed, and incubated as described below.

*Northern analysis.* Total RNA was prepared from BMM (2 × 10^5^/ml) incubated for 2 days in M-CSF (10 ng/ml) and then treated with M-CSF and combinations of RANKL and TGF-β for 24 h, with or without OPG (100 ng/ml), and anti TNF-α antibody (10 mg/ml) using a commercially available kit. Fifteen micrograms of total RNA was denatured, separated on a 1.2% agarose-formaldehyde gel, transferred to a Hybond–N membrane (Amersham International), and hybridized for 16 h at 42 °C with ^32^P-labeled cDNA probes for murine NFATc1, *c-fos*, and β-actin prepared by the random primer method. After hybridization membranes were washed at 42 °C (2× SSPE and 0.1% SDS; 1× SSPE and 0.1% SDS; 0.5× SSPE and 0.1% SDS) and autoradiographed using Hyperfilm.

*Quantitative RT-PCR.* BMM (2 × 10^5^/ml) were incubated for 2 days with M-CSF (10 ng/ml) and then treated with combinations of M-CSF (10 ng/ml), RANKL (30 ng/ml), TGF-β1 (0.4 ng/ml) or pan-specific TGF-β antibody (10 mg/ml) for 24 h. Total RNA was extracted from these cultures and reverse transcribed with M-MLV. Real-time PCR was performed on an I-cycler (Bio-Rad, UK) using the DNA-binding dye SYBR green for detection of PCR products. A total of 2 μl of external plasmid standard or cDNA was added to a final reaction volume of 25 μl containing 0.05 U/μl Taq, SYBR green, and specific primers (0.2 μM). Primers used were as follows: murine NFATc1 sense 5′-CCGTTGCTTCCAGAAAATAACA-3′; NFATc1 antisense, 5′-TGTGGGATGTGAACTCGGAA-3′; β-actin sense 5′-GTCATCACTATTGGCAACGAG-3′; and antisense 5′-CCTGTCAGCAATGCCTGGTACAT-3′. Reaction conditions were 95 °C for 3 min, followed by 35 cycles of 95 °C for 20 s, 59 °C for 20 s, and 72 °C for 20 s. For each sample NFATc1 mRNA levels were expressed as relative copy number normalized to 10° β-actin mRNA copies.

*Immunofluorescence.* BMM or RAW 264.7 cells were seeded onto glass coverslips and incubated in M-CSF (30 ng/ml) or RANKL (100 ng/ml) for 5 days to generate osteoclasts. Cells were washed in EMEM, incubated in M-CSF for 1 h to remove RANKL, and then stimulated with TGF-β (1 ng/ml) for 30 min. The cellular distribution of NFATc1 was assessed as follows. Coverslips were removed, washed in PBS, fixed in 4% paraformaldehyde, permeabilized with 0.1% Triton X-100, incubated with 1% goat serum, and incubated with a specific anti-mouse NFATc1 monoclonal antibody diluted 1:50 in 1% goat serum for 1 h. Cells were washed in PBS, incubated for 2 h with a biotinylated goat anti-mouse secondary (Vector Labs, USA), and then incubated for 2 h with fluorescein conjugated streptavidin (Vector Labs, USA). Fluorescence was visualized using a Leica HC microscope. Photographs were taken with a JVC digital camera linked to image pro-plus so at a magnification of 400× or 1000×.

*Assessment of osteoclast formation.* Osteoclast formation was evaluated using the specific osteoclast marker tartrate resistant acid phosphatase (TRAP) [14]. After incubation, cells were washed in PBS, fixed in 10% formalin, washed, and stained for TRAP. Cells were counterstained with hematoxylin and examined at 40× magnification on a light microscope fitted with an eyepiece graticule.

*Statistical analysis.* Differences between groups were assessed using ANOVA (Statview; Abacus concepts, USA). A difference of *p* < 0.05 was considered significant.

## Results and discussion

### TGF-β commits monocytes to the osteoclastic lineage within 24 h

To establish when TGF-β commits monocytes to the osteoclast lineage BMM were pre-incubated with TGF-β for 1–72 h, and then cultured with combinations of M-CSF, RANKL, and IFN-γ. BMM exposed to TGF-β for only part of the pre-incubation period were grown with M-CSF alone for the remainder of the pre-treatment. Following pre-treatment all cultures were incubated for 5 days with combinations of M-CSF, RANKL, TGF-β, and IFN-γ.

**Fig. 1:**
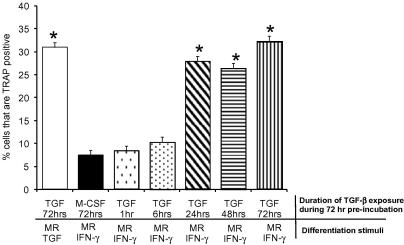
Pre-incubation with TGF-β for 24 h is sufficient to commit BMM to the osteoclast lineage. BMM were pre-treated with M-CSF with or without TGF-β for 1, 6, 24, 48, and 72 h to determine the minimum period sufficient for TGF-β to antagonize the anti-osteoclastic action of IFN-γ. All cultures were pre-treated for 72 h irrespective of whether they received TGF-β during pre-treatment regime. Cultures exposed to TGF-β for a proportion of the pre-incubation period had TGF-β removed after the relevant exposure time and replaced with M-CSF. Following pre-treatment all cultures were washed and incubated for 5 days with combinations of M-CSF, RANKL, TGF-β, and IFN-γ. Controls consisted of BMM incubated with TGF-β throughout the pre-incubation period and exposed to M-CSF, RANKL, and TGF-β in the differentiation period. Values are expressed as means ± SEM of four separate experiments *n* = 20, ^∗^*p* < 0.05 versus group pre-treated with M-CSF alone then cultured in M-CSF, RANKL, and IFN-γ.

As shown previously, IFN-γ suppressed RANKL-induced osteoclast formation (Fig. 1) [15]. Pre-incubation with TGF-β for 1–6 h was unable to overcome the inhibitory action of IFN-γ. Whereas, pre-incubation with TGF-β for 24 h completely abolished IFN-γ’s anti-osteoclastic action. Pre-incubating for 48 or 72 h did not further enhance osteoclast formation, suggesting that the intracellular events mediating TGF-β’s augmentative action are completely active within 24 h of TGF-β exposure. Therefore, TGF-β facilitates the initial events governing osteoclast formation.

### TGF-β induces NFATc1 expression within 24 h and enables RANKL induced NFATc1 expression

Binding of RANKL to RANK activates several transcription factors responsible for promoting osteoclastic gene expression. These are not all activated within the same time-frame: early-response factors, such as *c-fos*, are activated before late-response factors, such as NFATc1 [16]. Like TGF-β, these are essential for osteoclast differentiation. Precursors lacking NFATc1 or *c-fos* will not undergo osteoclast formation [12,13], whereas ectopic NFATc1 expression under certain circumstances may be sufficient for osteoclast differentiation in the absence of activating cytokines [17]. NFATc1 is therefore critical for osteoclastogenesis.

**Fig. 2:**
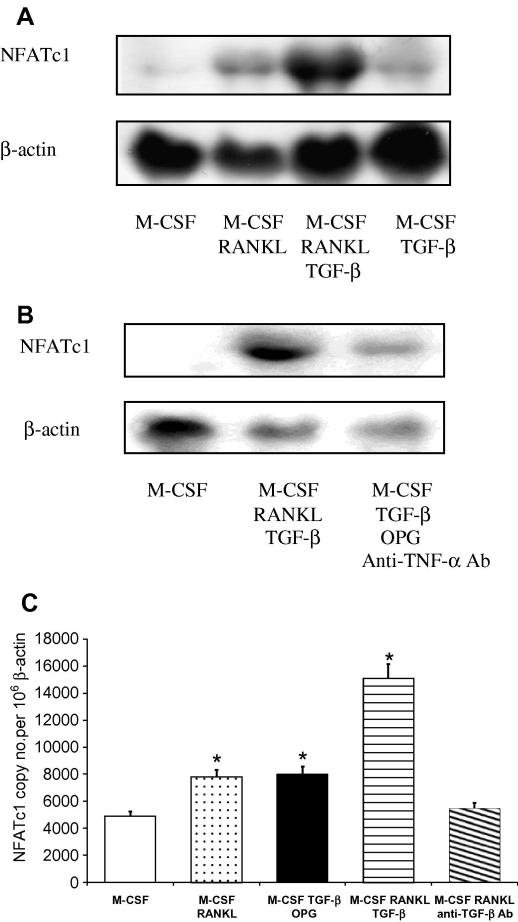
TGF-β directly induces NFATc1 mRNA expression in BMM within 24 h and enables RANKL-induced NFATc1 expression. (A) Total RNA was prepared from BMM incubated with M-CSF and combinations of RANKL and TGF-β for 24 h and Northern analysis performed for NFATc1 and β-actin. (B) Total RNA was isolated from BMM incubated with M-CSF and combinations of TGF-β with or without OPG and anti-TNF-α antibody for 24 h. Northern analysis was then performed for NFATc1 and β-actin. (C) BMM were incubated in M-CSF for 2 days. Cultures were treated with M-CSF and combinations of RANKL and TGF-β, with or without OPG or anti-TGF-β antibody for 24 h. Total RNA was extracted and analyzed using quantitative RT-PCR. Results are expressed as NFATc1 copy number per 10^6^ β-actin and are means of three separate experiments ± SEM, ^∗^*p* < 0.05 versus all groups.

In light of this, we examined the effect of TGF-β on NFATc1 expression. NFATc1 mRNA expression was low in non-committed BMM, whereas RANKL induced detectable levels within 24 h, which was strongly augmented in the presence of TGF-β (Fig. 2A). Furthermore, TGF-β itself induced a significant increase in NFATc1 expression (Fig. 2A and C) even in the presence of OPG and anti-TNF-α antibody (Fig. 2B). This suggests that TGF-β is able to directly induce NFATc1 and also enhance RANKL-induced NFATc1 expression during the time-period when it enables and augments osteoclast differentiation.

TGF-β and NFATc1 are both essential for osteoclastogenesis, as sequestering TGF-β suppresses RANKL-induced osteoclast differentiation [5], while RANKL stimulated osteoclastogenesis is impaired in NFATc1 deficient monocytes [18]. Thus, it is conceivable that TGF-β’s facilitative action may depend on its ability to regulate RANKL-induced NFATc1 expression during initial stages of osteoclast differentiation. To assess this, the ability of RANKL to induce NFATc1 was assessed in cultures in which TGF-β was removed using pan-specific TGF-β antibody. This prevented RANKL-induced NFATc1 expression, with levels not significantly differing from M-CSF-treated controls (Fig. 2B and C), suggesting that RANKL-induced NFATc1 expression, a critical step in the molecular events determining osteoclast formation, is dependent on TGF-β’s enabling action and without this input osteoclast formation cannot occur.

This dependence could represent one of two possibilities. First, it could reflect the established requirement of TGF-β for osteoclast differentiation; the lack of expression occurring as a secondary consequence of the block in osteoclast formation. On the other hand, it could be the primary mechanism by which TGF-β enables osteoclastogenesis: TGF-β providing an input to induce NFATc1 expression in non-committed precursors. NFATc1 is thereafter available to be activated by RANKL to propagate the chain of downstream events that result in osteoclast formation. At present, it is not possible to draw a firm conclusion on which interpretation is correct; however, the ability of TGF-β to directly stimulate NFATc1 expression is more in keeping with the second hypothesis.

### TGF-β does not induce *c-fos* expression or NFATc1 nuclear translocation

While TGF-β induces NFATc1 expression it is unable to stimulate osteoclastogenesis. Previous studies suggest that NFATc1 is not only necessary for osteoclast formation but may be sufficient to stimulate osteoclastogenesis on its own [19]. If true, then it raises the question of why TGF-β does not stimulate osteoclastogenesis in the absence of additional activating stimuli. There are several explanations for this; first TGF-β may not induce all the transcription factors that cooperate with NFATc1 to promote osteoclastic gene expression such as members of the AP-1 family [12,18]. One key AP-1 member involved in osteoclast differentiation is *c-fos*, which is induced in monocytes by RANKL [20]. However, the effect of TGF-β on *c-fos* expression is unclear. Second, while TGF-β induces NFATc1 expression it may not provide the necessary inputs to stimulate its nuclear translocation. This is regulated by post-translational modifications that expose nuclear localization signals [21]. Without these modifications osteoclastic gene expression cannot proceed [12,19,22]. Thus, it is conceivable that while TGF-β induces NFAT expression it may not necessarily stimulate nuclear translocation.

**Fig. 3:**
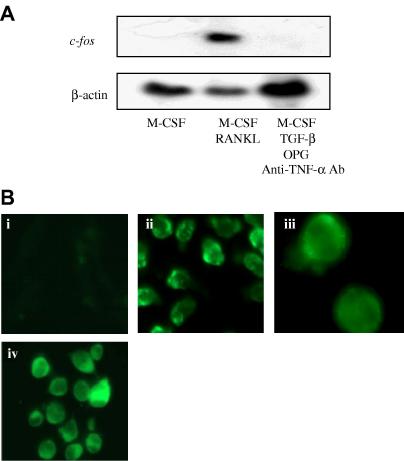
TGF-β does not induce *c-fos* expression or nuclear translocation of NFATc1. (A) Total RNA was prepared from BMM incubated with M-CSF; M-CSF, and RANKL or M-CSF, RANKL, TGF-β, OPG, and anti-TNF-α neutralizing antibody for 24 h. Northern analysis was then performed for NFATc1 and β-actin. (B) Immunolocalization of NFATc1 i, BMM incubated with M-CSF; magnification 40×. ii and iii, BMM treated with M-CSF and TGF-β for 45 min; magnification 40× and 100×. iv, RAW264.7 cells treated with TGF-β for 45 min; magnification 40×.

Therefore, to assess these possibilities we examined the ability of TGF-β to induce *c-fos* expression and NFATc1 nuclear translocation. To examine *c-fos* expression BMM were incubated for 24 h with M-CSF and TGF-β in the presence of OPG and anti-TNF-α antibody to sequester any RANKL or TNF-α present in the culture environment. *c-fos* mRNA was not detectable in control cultures treated with M-CSF alone, but was readily detectable in cultures incubated with M-CSF and RANKL for 24 h (Fig. 3A). However, in contrast to its action on NFATc1, TGF-β was unable to induce *c-fos* expression in BMM (Fig. 3A). To examine the ability of TGF-β to regulate NFATc1 migration, fluorescent immunocytochemistry was used to determine the distribution of NFATc1 in BMM treated with or without TGF-β. In keeping with the Northern analysis data, NFATc1 immunofluorescence was not detected in unstimulated BMM (Fig. 3Bi), whereas BMM or RAW264.7 cells incubated with TGF-β displayed intense NFATc1 staining (Fig. 3Bii–iv). Staining was predominantly cytoplasmic, suggesting that while TGF-β increases NFATc1 protein levels it is not sufficient to induce nuclear translocation. Therefore, although TGF-β may be required for NFATc1 expression it is not sufficient to induce its activation and nuclear translocation in monocytes. This suggests that the inability of TGF-β to stimulate osteoclastogenesis is due to a combination of failing to induce *c-fos* and activate NFATc1 nuclear translocation.

### TGF-β is responsible for NFATc1 expression during TNF-α-induced osteoclastogenesis

Other cytokines in addition to RANKL are also capable of activating osteoclast formation. One such factor, TNF-α, directly stimulates osteoclast formation from BMM [23], and may have a role in excessive osteoclast formation seen in post-menopausal osteoporosis and rheumatoid arthritis [24]. TGF-β is also essential for TNF-α-induced osteoclastogenesis [4]. TNF-α binding to its receptor activates a cascade of intracellular signals but in contrast to RANKL it does not activate TRAF6 which is required for the initial induction of NFATc1 expression [12]. As NFATc1 is critical for the early stages of osteoclast differentiation and TGF-β is able to directly induce NFATc1 expression within 24 h we examined the possibility that TGF-β-induced NFATc1 expression has a role in TNF-α activated osteoclast formation.

**Fig. 4:**
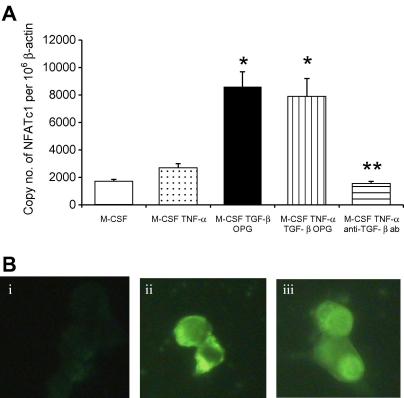
TGF-β facilitates NFATc1 expression in TNF-α-induced osteoclasts. (A) BMM were incubated in M-CSF for 2 days. Cultures were treated with M-CSF and combinations of TNF-α, TGF-β, pan-specific neutralizing TGF-β antibody, and OPG for 24 h. Total RNA was extracted and NFATc1 expression analyzed using quantitative real-time PCR. Results are means of separate experiments expressed as NFATc1 copy number per 10^6^ β-actin ± SEM. ^∗^*p* < 0.05 versus M-CSF, M-CSF plus RANKL, and M-CSF, RANKL plus TGF-β antibody. (B) Immunolocalization of NFATc1 in RAW264.7 cells incubated for 48 h in TNF-α (i), TGF-β (ii) or TGF-β for 48 h followed by 45 min with TNF-α (iii).

TNF-α did not significantly increase NFATc1 expression in monocytes, levels not differing from M-CSF controls (Fig. 4A). In contrast, TGF-β directly induced a significant 4.6-fold increase in NFATc1 expression within 24 h (Fig. 4A), and similar levels were noted in cultures incubated with both TGF-β and TNF-α (Fig. 4A). Thus, the inability of TNF-α to induce NFATc1 suggests that another TGF-β may enable TNF-α-induced osteoclast formation by providing NFATc1 during the initial stages of differentiation. This is further underlined by a 2.9-fold reduction in NFATc1 expression in TNF-α treated cultures incubated with pan-specific TGF-β antibody (*p* < 0.05), suggesting that basal levels of NFATc1 expression are to a large extent attributable to TGF-β present within the culture environment (Fig. 4A). The inability of TNF-α to induce NFATc1 expression without TGF-β being present was confirmed in immunofluorescence studies (Fig. 4B). No appreciable difference in staining intensity was seen in control cultures compared with those treated with TNF-α (Fig. 4Bi and ii). However, while TNF-α is unable to induce NFAT expression, it is able to stimulate nuclear accumulation of NFATc1 in cultures that have been pre-treated with TGF-β (Fig. 4Biii). TNF-α activated osteoclast differentiation would therefore appear to rely on TGF-β to promote an initial cytoplasmic increase in NFATc1, which is subsequently translocated to the nucleus under the influence of activating stimuli provided by TNF-α. Therefore, the formation of osteoclasts in inflammatory conditions such as rheumatoid arthritis may be controlled by a reciprocal interaction between TGF-β and TNF-α on NFATc1 expression and activity; TGF-β promoting cytoplasmic accumulation of NFATc1 which subsequently translocates to the nucleus under the influence of TNF-α.

To conclude our data clearly show that TGF-β influences osteoclast formation by regulating NFATc1 expression, a critical component of the osteoclastic transcriptional machinery. TGF-β acts directly on non-committed monocytes to enhance RANKL-induced NFATc1 expression; moreover, RANKL is unable to induce NFATc1 in the absence of TGF-β. Thus, both the augmentative and enabling action of TGF-β on osteoclast formation may be mediated through its ability to control cytoplasmic levels of NFATc1 during the early stages of osteoclastogenesis. Furthermore, it is likely that TGF-β may facilitate TNF-α-induced osteoclast formation through a very similar NFATc1-dependent mechanism. Formation of osteoclasts during physiological remodeling and inflammatory conditions may be dependent on a coordinated interaction between TGF-β and RANKL/TNF-α, which regulates the expression and intracellular distribution of NFATc1 during the initial stages of osteoclastogenesis.
